# A guideline for placement of an infra-acetabular screw based on anatomic landmarks via an intra-pelvic approach

**DOI:** 10.1186/s13018-018-0786-1

**Published:** 2018-04-10

**Authors:** Florian Baumann, Paul Schmitz, Daniel Mahr, Maximilian Kerschbaum, Axel Gänsslen, Michael Nerlich, Michael Worlicek

**Affiliations:** 1 Department of Trauma Surgery, Regensburg University Medical Center, 93042 Regensburg, Germany; 2 Clinic for Trauma Surgery, Orthopedics and Hand Surgery, Klinikum Wolfsburg, Wolfsburg, Germany

**Keywords:** Acetabulum fracture, Screw fixation, Entry point, Infra-acetabular screw, Anatomic landmarks, Intra-pelvic approach

## Abstract

**Background:**

Due to demographic changes, more and more fracture patterns involving anterior acetabular structures occur. The infra-acetabular screw is seen a useful tool to increase stability in fixation of the acetabular cup. However, the exact position of this screw in relation to anatomic landmarks which are intra-operatively palpable via an intra-pelvic approach has not yet been determined.

**Methods:**

This biomorphometric experimental study references the ideal screw position of an infra-acetabular screw to anatomic landmarks palpable via an intra-pelvic approach. Therefore, we created a computer tomography-based 3D-model of 40 patients (20 women, 20 men) who received a computer tomography (CT) scan of the pelvis for any other reason than an acetabular fracture.

**Results:**

The entry point of an ideal infra-acetabular was of high constancy. At mean, this point was 10.2 mm caudal and 10.4 mm medial of the ilio-pubic/ilio-pectineal eminence. This reference is independent of age, gender, or physical dimensions. However, we found gender-dependent differences for the angulation and the length of the screw.

**Conclusions:**

This study provides a comprehensive guideline to determine the ideal entry point for an infra-acetabular screw via an intra-pelvic approach. The entry point is located 10.2 mm caudal and 10.4 mm medial of the ilio-pubic/ilio-pectineal eminence.

**Trial registration:**

Clinical Trial Registry University of Regensburg Z-2017-0930-1. Registered 04. Dec 2017.

## Background

Treatment of fractures of the acetabulum is one of the most challenging procedures in trauma surgery. Achieving an anatomic reduction of the joint surface can be demanding. However, it is necessary to establish a rigid fixation to retain the position until the fracture has healed.

**Fig. 1:**
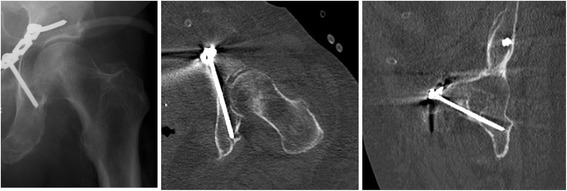
Radiographs showing an ideal position of the infra-acetabular screw

Large register studies on the epidemiology of acetabular fractures have shown an increasing number of cases with involvement of the anterior acetabular column during the last 20 years [19]. Therefore, anterior approaches and anterior operating techniques have been advanced [2, 3, 8, 10, 12, 17]. A dissociation of the anterior column to the posterior column or dislocation of the quadrilateral surface has been identified as a risk factor for a secondary dislocation leading to a central subluxation of the femoral head [3, 23, 24]. Several concepts to support the medial acetabular border have been introduced [4, 5, 11, 14, 18, 21, 24]. The infra-acetabular screw following Letournel’s [16, 17] concept of a peri-acetabular frame was described in 2011 by Culemann et al. [5]. Culemann et al. determined the entry point 1 cm caudal to the ilio-pubic/ilio-pectineal eminence (IPE) in the middle of the pubic ramus and stated that this screw was only applicable through an ilio-inguinal approach [5]. However, the intra-pelvic approach has become the working horse for acetabulum fractures today [2, 12]. Over the past years, experience and capabilities with this approach have been extended [7]. Hence, clinical experience has proven that the entry point and path of entry can easily be exposed. However, the exposure of the lateral border of the pubic rim is not as good as with the ilio-inguinal approach, and therefore, the instructions given by Culemann et al. for an ideal screw position (Fig. 1) are difficult to follow via an intra-pelvic approach [1, 5, 13, 17].

The purpose of the study is to specify the ideal position of the infra-acetabular screw in relation to anatomic landmarks that are accessible via an intra-pelvic approach.

## Methods

The Ethics Committee at the University of Regensburg approved the study in January 2018 (Institutional Review Board Number 17-705649-180). The study is registered at the Clinical Trial Registry University of Regensburg Z-2017-0930-1. All procedures performed in studies were in accordance with the 1964 Helsinki declaration.

This study is an experimental computer tomography-based 3D-model analysis to reference the ideal screw position of an infra-acetabular screw to anatomic landmarks. We investigated 3D-computer tomography (CT) scans of 40 patients (20 women, 20 men) obtained by the Department of Radiology of our institution.

Baseline characteristics of the study group are shown in Table 1. CT measurements were carried out using the “semi-automatic” function of a digital 3D CT-based planning software (Modicas, Erlangen, Germany). This mode offers the possibility to assess the pelvis in three dimensions, to exactly determine the axes, and to automatically calculate angles and to measure distances. First, the pelvis was virtually aligned in order to bring the anterior pelvic plane (APP) in congruence with the coronal plane, to have a constant starting point. The APP was defined as the triangle between the pelvic symphysis and both spinae iliacae anteriores superiores. The pelvic inlet plane was identified by the angle point of the pelvic brim at level of the acetabulum on both sides and the cranial margin of the pubic symphysis.

**Table 1:** Measurement results for the ideal position of an infra-acetabular screw

*N* = 40	Female	Male	Level of significance female compared to male patients	Total
Distance medial IPIS (in mm)	10.6 ± 1.22 (8.5–14.1)	10.2 ± 0.73 (8.6–12.0)	*p* = 0.770	10.4 ± 1.02 (8.5–14.1)
Distance caudal IPIS (in mm)	10.2 ± 1.61 (3.0–13.2)	10.2 ± 1.07 (8.4–13.7)	*p* = 0.883	10.2 ± 1.36 (3.0–13.70)
Sagittal angle (α) to PIP (in degrees)	9.6 ± 1.16 (6.9–12.4)	11.2 ± 1.57 (8.8–15.1)	*p* = 0.000	10.4 ± 1.58 (6.9–15.10)
Axial angle (β) to PIP (in degrees)	71.4 ± 3.55 (66.7–89.4)	69.5 ± 2.42 (62.3–74.3)	*p* = 0.006	70.5 ± 3.17 (62.3–89.4)
Screw length (in mm)	74.8 ± 3.4 (70.20–84.00)	83.9 ± 2.05 (79.80–88.30)	*p* = 0.000	79.3 ± 5.37 (70.2–88.30)

**Fig. 2:**
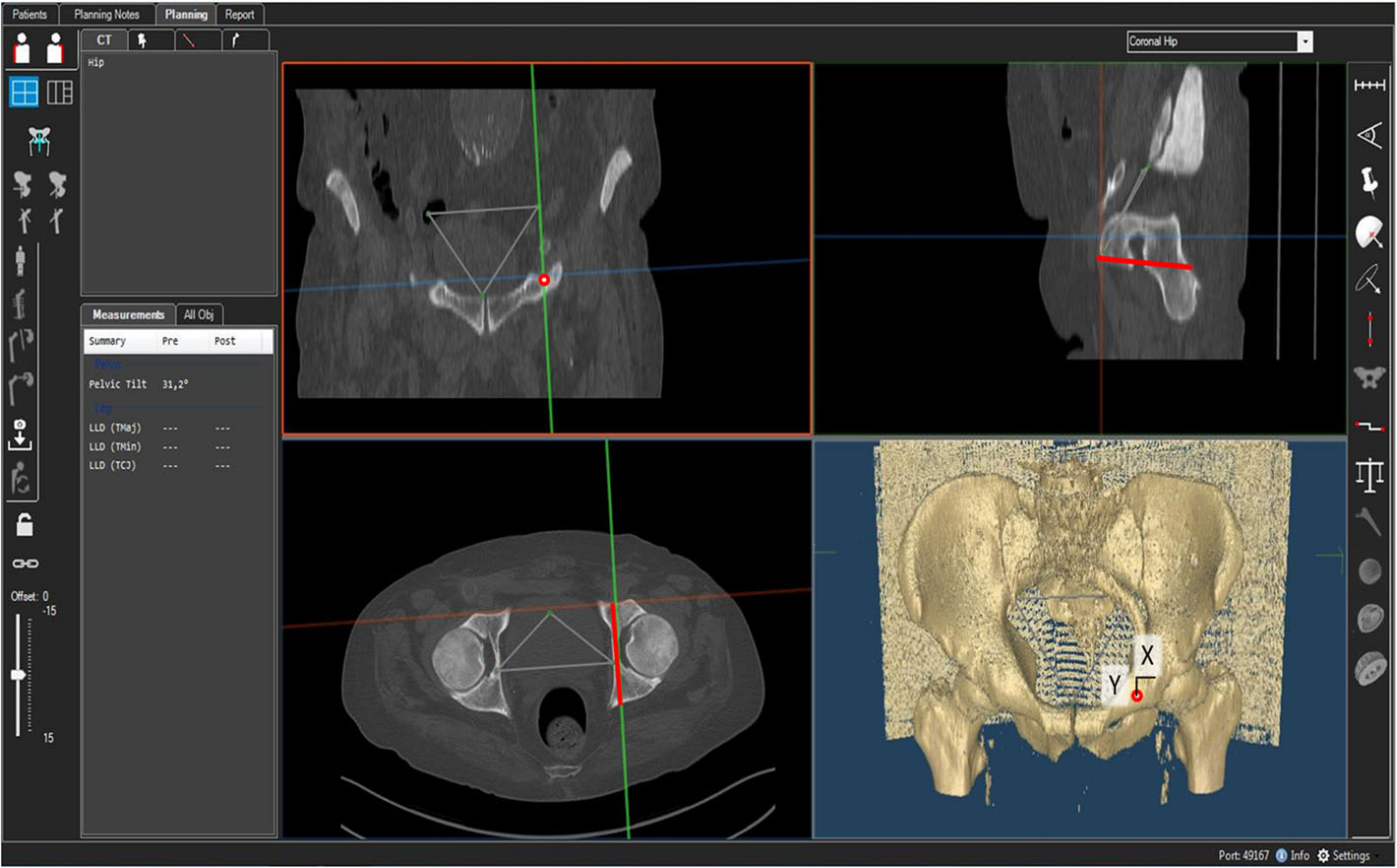
Setting of the 3D-CT-based measurement of the infra-acetabular screw position

The optimal position of the infra-acetabular screw was identified by locating the infra-acetabular corridor on axial views of the CT. The first point of the straight line through the infra-acetabular corridor was defined by the center of the acetabular fossa, 2 mm lateral to the quadrilateral surface. Then, the straight line was placed parallel to the quadrilateral surface and symmetrically along the infra-acetabular corridor into the ischial cortex. After determination of the optimal position of the screw, the orientation of the screw was defined by measuring the sagittal (α) and axial (β) angles in relation to the pelvic inlet plane. The entry point of the straight line was referenced by measuring the relation to the apex of the IPE. Therefore, the distance was measured on a horizontal line (*x*) and on a perpendicular (*y*) line (Fig. 2).

### Statistical analysis

Statistical analysis was performed using the software package SPSS (Version 23, SPSS Inc., Chicago, IL).

Since there is no previous data on the variance of the entry point, this preliminary study was designed as an exploratory pilot study without any a priori sample size calculation based on a primary endpoint. Based on the variance of the angle related to the anterior pelvic plane, a sample size of 40 patients was considered feasible and expected to have enough power.

For comparison of mean values, we used the independent *t* test. The Spearmen’s rank correlation was used to investigate a relationship between age, height, and weight of the patient. Correlation coefficients ≥ .40 indicate a relevant relationship. Unless otherwise stated, descriptive data are given as mean ± standard deviation. The level of significance was at *p* < 0.05 for all tests.

## Results

**Fig. 3:**
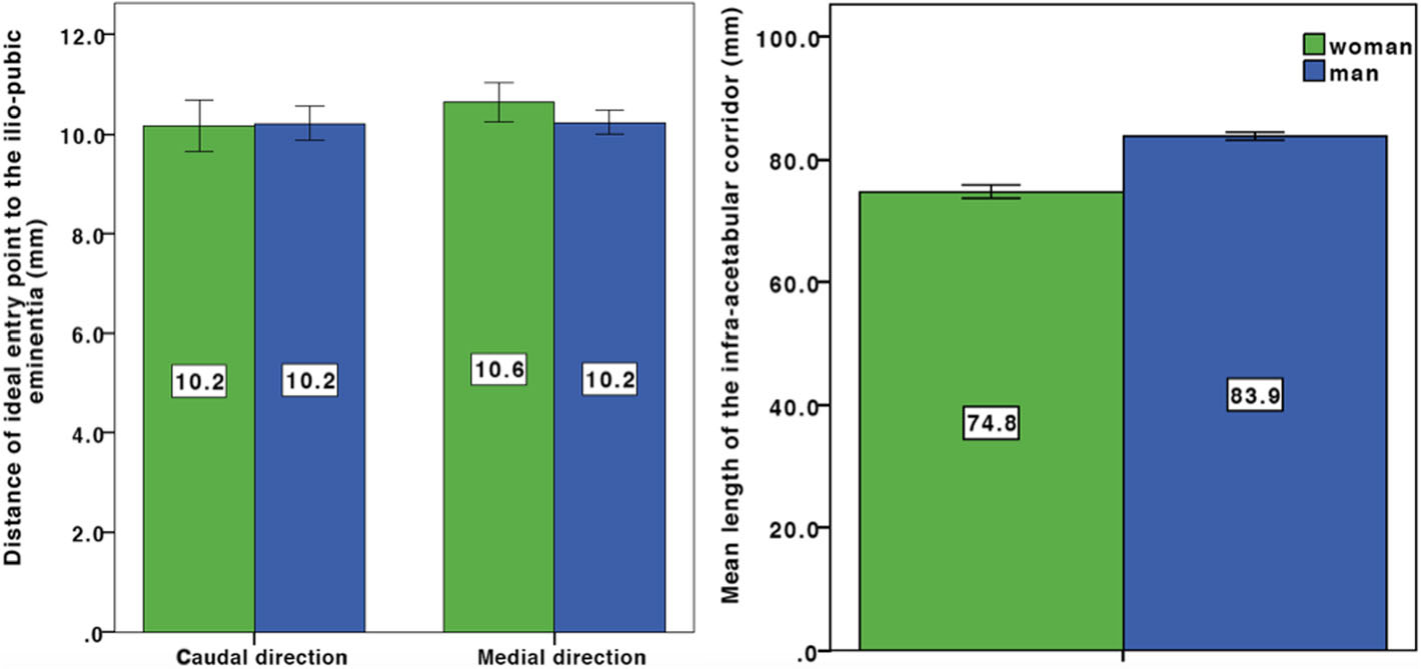
Boxplot of the distance of the ideal entry point to the ilio-pubic/ilio-pectineal eminence and the screw length

Table 1 shows baseline data recorded in 40 patients. The mean entry point for an ideal infra-acetabular screw was 10.2 mm (± 1.4) caudal and 10.4 mm (± 1.0) medial to the IPE. There was also no significant difference for the position of the entry point between male and female patients (Fig. 3a). The mean length of the infra-acetabular corridor was 79.2 mm (± 5.3) (Fig. 3b). We found a significant difference between male and female patients concerning the length of the corridor. The mean length in female patients was 74.8 mm (± 3.4) and in male patients, 83.9 mm (± 2.1) (*p* = 0.000) respectively.

**Fig. 4:**
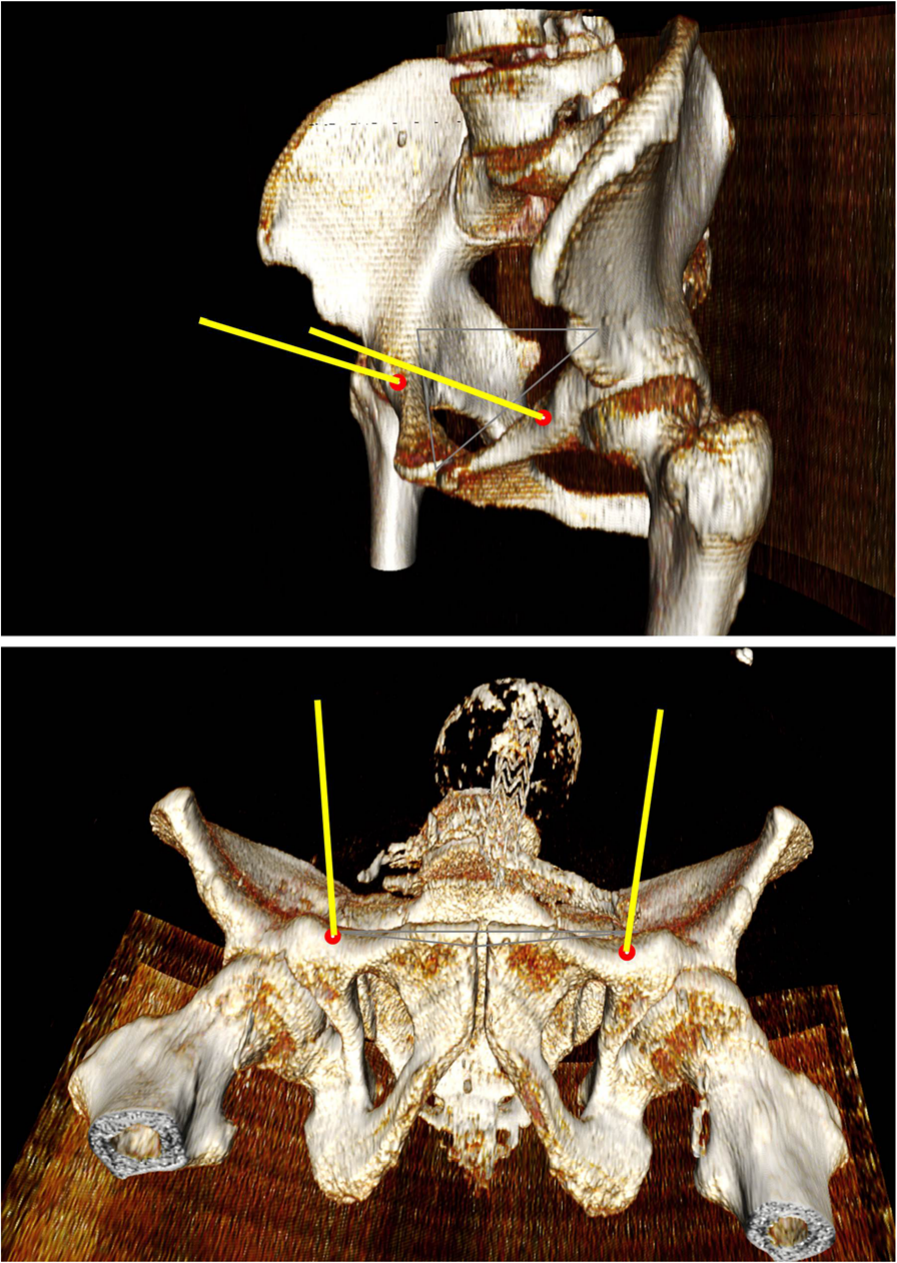
3D-CT virtual reality images illustrating the angle of the drill of left and right screw by lines in relation to the PIP

The mean angle of both left and right screw related to the axial plane was 10.4° (± 1.6°) and to the sagittal plane 70.5° (± 3.2). The mean angle in female patients related to the axial plane was 11.2° (± 1.6) and to the sagittal plane 71.4° (± 3.6). Male patients had a mean angle of 9.6° (± 1.2°) related to the axial plane and 71.4° (± 3.6) to the sagittal plane. This differences between female and male patients were significant (*p* = 0.006 for the sagittal and *p* = 0.000 for the axial plane). We did not find any significant difference between the right and left side concerning the screw position, length of the corridor, or angulation. Figure 4 shows 3D-CT virtual reality images illustrating the angle of the drill of the left and right screw by lines in relation to the proximal interphalangeal (PIP).

## Discussion

The most important finding of the present study is that the ideal entry point for an infra-acetabular screw is 10.2 mm caudal and 10.4 mm medial of the IPE. This is the first study to report on the ideal screw position in relation to directly palpable landmarks via an intra-pelvic approach. Additionally, we referenced the angle of the screw to the pelvic inlet plane which is visible intraoperatively. This referencing in relation to visible anatomic landmarks may allow the surgeon to reproducibly place the infra-acetabular screw in an optimal position.

Demographic changes lead to a rising number of geriatric acetabular fractures involving anterior acetabular structures (anterior wall/anterior column) [6, 19, 20, 23]. Baseline data of our patient population regarding age and physical dimensions reflect the characteristics of a typical patient population for fractures involving anterior acetabular elements. This is concordant with other studies on the configuration of the infra-acetabular corridor [5, 9].

Historically, the posterior Kocher/Langenbeck approach was the standard approach to stabilize acetabular fractures [13, 16]. During the last 20 years, fracture patterns have changed so that anterior approaches are used most frequent today [8]. This trend initiated further advancement of anterior approaches and operative techniques via anterior approaches [7, 11, 14, 18, 21, 24]. There is an ongoing discussion about the “safe zones” and “dangerous zones” in acetabular surgery [25]. Due to the variance in pelvic anatomy, the safe zones are often relatively far away from the acetabulum, making it difficult to obtain adequate peri-acetabular stability. In 2011, Culemann et al. [5] proposed a modified quadrilateral screw inserted in the region of the Koehler’s teardrop. This so-called *infra-acetabular* screw was intended to minimize the risk of intra-articular placement of the screw. The entry point for this modified screw was reported to be 1 cm caudal to the IPE and in the middle of the pubis ramus. When this infra-acetabular screw position was introduced, the anterior ilio-inguinal approach which allows direct access to the pubic ramus circumference was the most frequent anterior approach [19]. However, identification of the middle of the pubic ramus can be difficult via an intra-pelvic approach, which is the standard anterior approach today [8]. Therefore, the instructions to identify the ideal entry point determined by Culemann et al. [5] are not applicable via the intra-pelvic approach. We investigated the ideal screw position in CT-based 3D-models and determined the relation to anatomic landmarks which are intra-operatively palpable via an intra-pelvic approach. We indicate a reproducible guideline for the placement of an infra-acetabular screw via an intra-pelvic approach.

The major advantage of the intra-pelvic approach is a direct inspection of the quadrilateral surface which is often involved in geriatric fractures [1–3, 8, 10, 19, 25]. In cases of reduced bone quality, a simple fall can cause a fracture of the anteromedial acetabulum or the quadrilateral surface by load transmission through the major trochanter [4]. A lack of support of the quadrilateral surface has been identified as a risk factor for a secondary dislocation leading to a central subluxation of the femoral head [6, 11, 20, 24]. In recent years, different concepts were introduced to increase medial support of the quadrilateral surface to prevent this complication [3–5, 7, 10, 11, 18, 21, 24].

Letournel was the first to describe the construct of a peri-acetabular frame to improve stability in acetabular fracture fixation [16]. They recommended a screw placement through the acetabular fossa. Culemann et al. [5] modified the position of this quadrilateral surface screw to the infra-acetabular corridor to minimize the risk of an intra-articular placement of the screw. The diameter of the drill hole for the infra-acetabular screw is 2.5 mm. The standard 3.5-mm fully threaded screw does not apply any inter-fragmentary compression force to the fracture and, therefore, only works as a distance screw. However, the main functions of this screw are a fixation of the anterior column to the posterior column and to prevent a dislocation of the quadrilateral surface [5, 18]. Gras et al. [11] found in a biomechanical cadaveric study that an additional placement of an infra-acetabular screw significantly increases the stability of the fracture fixation compared to a standard plate fixation.

Recently, a large biomorphometric CT-based study identified a viable infra-acetabular corridor with a diameter over 5 mm in 93% of specimens [9]. This study measured the entry point in relation to the pubic symphysis. The pubic symphysis is well-accessible via an intra-pelvic approach. However, they found a large variance for this distance of 54–91 mm [9]. This variance is due to variation of the configuration of the pubic rim and makes this reference not useful for clinical practice. This study measured the angle of the screw to the anterior pubic plane. The APP is well-established in the determination of the pelvic tilt in hip arthroplasty [15]. It can be identified in a prone position and provides accurate and reproducible referencing plane in 3D-analyses. However, several studies have demonstrated an impairment in clinical reproducibility of the APP leading to inaccuracy of the implant position [22]. We intended to orientate the angle of the screw to a plane that is clearly visible for the surgeon via an intra-pelvic approach. Therefore, we chose the pelvic inlet plane as the reference plane which can be easily identified by the ilio-pubic brim and the symphysis. We could confirm the sex-specific differences in angulation of the screw reported by Gras et al. [9]. We found a mean length of 75 mm in female and 84 mm in male patients respectively. The screw length in our patient population was therefore somewhat shorter than that in other studies [5, 9].

This study has some limitations. The guideline for placement of the infa-acetabular screw is based on the identification of the IPE. This anatomic landmark is a large-area bony bump rather than a sharply marked spike. However, via an intra-pelvic approach, it is well-palpable and its tip can easily be identified [25]. Another limitation to transfer this guideline into clinical practice is an insufficient reduction of the fracture. Incongruence of the infra-acetabular corridor can lead to narrowing of the designated screw canal or even to occlusion of the safe zone for screw placement. An intra-articular screw position may be the consequence. Therefore, an accurate radiographic review of the screw position is mandatory. Lastly, the limited number of cases reduces the generalizability of the guideline.

## Conclusion

This study provides a comprehensive guideline to determine the ideal entry point for an infra-acetabular screw via an intra-pelvic approach. The entry point is located 10.2 mm caudal and 10.4 mm medial of the IPE. This reference is independent of age, gender, or physical dimensions. However, we found gender-dependent differences for the angulation and the length of the screw. Further prospective studies are needed to the proof effectiveness of this guideline in a clinical setting.

## Abbreviations

CT: Computer tomography
IPE: Ilio-pubic/ilio-pectineal eminence
